# Barriers to equitable maternal health in Aotearoa New Zealand: an integrative review

**DOI:** 10.1186/s12939-019-1070-7

**Published:** 2019-10-30

**Authors:** Pauline Dawson, Chrys Jaye, Robin Gauld, Jean Hay-Smith

**Affiliations:** 1 Department of Women’s and Children’s Health, Dunedin School of Medicine, University of Otago, Dunedin, New Zealand; 2 Department of General Practice and Rural Health, Dunedin School of Medicine, University of Otago, Dunedin, New Zealand; 3 Otago Business School, University of Otago, Dunedin, New Zealand; 4 Centre for Health Systems and Technology, University of Otago, Dunedin, New Zealand; 5 Rehabilitation Teaching and Research Unit, University of Otago, Wellington, New Zealand

**Keywords:** Maternal health, Health equity, New Zealand, Social determinants of health

## Abstract

**Background:**

The purpose of this review was to examine the literature for themes of underlying social contributors to inequity in maternal health outcomes and experiences in the high resource setting of Aotearoa New Zealand. These ‘causes of the causes’ were explored and compared with the international context to identify similarities and New Zealand-specific differences.

**Method:**

A structured integrative review methodology was employed to enable a complex cross disciplinary analysis of data from a variety of published sources. This method enabled incorporation of diverse research methodologies and theoretical approaches found in the literature to form a unified overall of the topic.

**Results:**

Six integrated factors – Physical Access, Political Context, Maternity Care System, Acceptability, Colonialism, and Cultural factors – were identified as barriers to equitable maternal health in Aotearoa New Zealand. The structure of the maternal health system in New Zealand, which includes free maternity care and a woman centred continuity of care structure, should help to ameliorate inequity in maternal health and yet does not appear to. A complex set of underlying structural and systemic factors, such as institutionalised racism, serve to act as barriers to equitable maternity outcomes and experiences. Initiatives that appear to be working are adapted to the local context and involve self-determination in research, clinical outreach and community programmes.

**Conclusions:**

The combination of six social determinants identified in this review that contribute to maternal health inequity is specific to New Zealand, although individually these factors can be identified elsewhere; this creates a unique set of challenges in addressing inequity. Due to the specific social determinants in Aotearoa New Zealand, localised solutions have potential to further maternal health equity.

## Background

Health inequity is typically measured and demonstrated by differences in health status, health outcomes, and experiences of health care, across groups of people that are demographically distinct. While region and author define inequity differently [1–5], for the purposes of this study, we follow Whitehead’s landmark work and define inequity as “differences in health which are not only unnecessary and avoidable but, in addition, are considered unfair and unjust” (p.5 [2]).

In seeking to identify the reasons for differences in health status and outcome, much research looks to the clinical causes, e.g. the reasons why a demographically diverse group who share the same condition may not achieve the same improvements in health when offered the same treatment. While differences in outcomes are frequently attributed to clinical factors, such as co-morbidities, the Commission on the Social Determinants of Health [3] is unequivocal that the underlying causes of health inequity are social, e.g. not everyone has the same capability to take advantage of provided opportunities [4, 5].

Maternal health inequity is most apparent when a global comparison is made between developing and developed countries and it is almost inevitable that outcomes may be poorer in low income countries than higher ones [6]. However, not all countries within an income grouping achieve the same outcomes. For example, the USA has the highest per capita health spend [7], and yet in 2015 had a maternal mortality rate of 26.4 per 100,000 compared to 7.3 per 100,000 of its near neighbour Canada [8]. It should be noted that these variations may be due to differences in age groups, fertility rate and population between these countries as well as financial constraints. Furthermore, within countries there can be large variations. For example, in Australia in the 2008–2012 period, the Aboriginal and Torres Strait Islander Maternal Mortality Rate (MMR) was 13.8% compared with 6.6% for non-Indigenous Australian women who gave birth fully funded [9]. This suggests that inequity in maternal health is dependent on location, population, cultural and societal context, and organisation of maternity systems.

The focus of this structured integrative critical review is the underlying social contributors to inequity in maternal health in Aotearoa New Zealand (hereafter, New Zealand). Relevant literature was examined for evidence of inequity arising from the ‘causes of the causes’ – “the fundamental global and national structures of social hierarchy and the socially determined conditions these create in which people grow, live, work, and age.” (p.42 [3]) The aim was to identify the New Zealand specific determinants – comparing these with determinants identified in global maternal health inequity literature – to understand inequity in our local context of a dispersed rural-urban multi-ethnic population mix (New Zealand had a population of 4.693 million people in 2016 spread over a land area of 268,021 km^2^, which is just larger than the United Kingdom at 242,495 km), and a publicly-funded health care system where maternity services are fully funded and at no cost to women and their families.

### Maternal inequity in New Zealand

Essentially, the New Zealand health system is a socialised, universal health care system [10], with maternity care free to all New Zealand resident women, from primary to tertiary level, unless a woman chooses to pay for a private obstetrician. A key point of difference of the New Zealand maternity service from many other countries is that the majority of women (93.6% in 2015 [11]) are cared for by autonomous, self-employed midwives who are contracted to the state [12, 13].

This midwifery-led continuity model is often promoted as the gold standard for maternity care for low risk women [14] who report fewer interventions and higher satisfaction than with other models such as medical-led or shared care [15]. It is also touted as a major contributor to the reduction of inequity [16, 17], even so, maternal health inequity persists in outcomes, care processes, and women’s experience within the New Zealand health system.

**Fig. 1:**
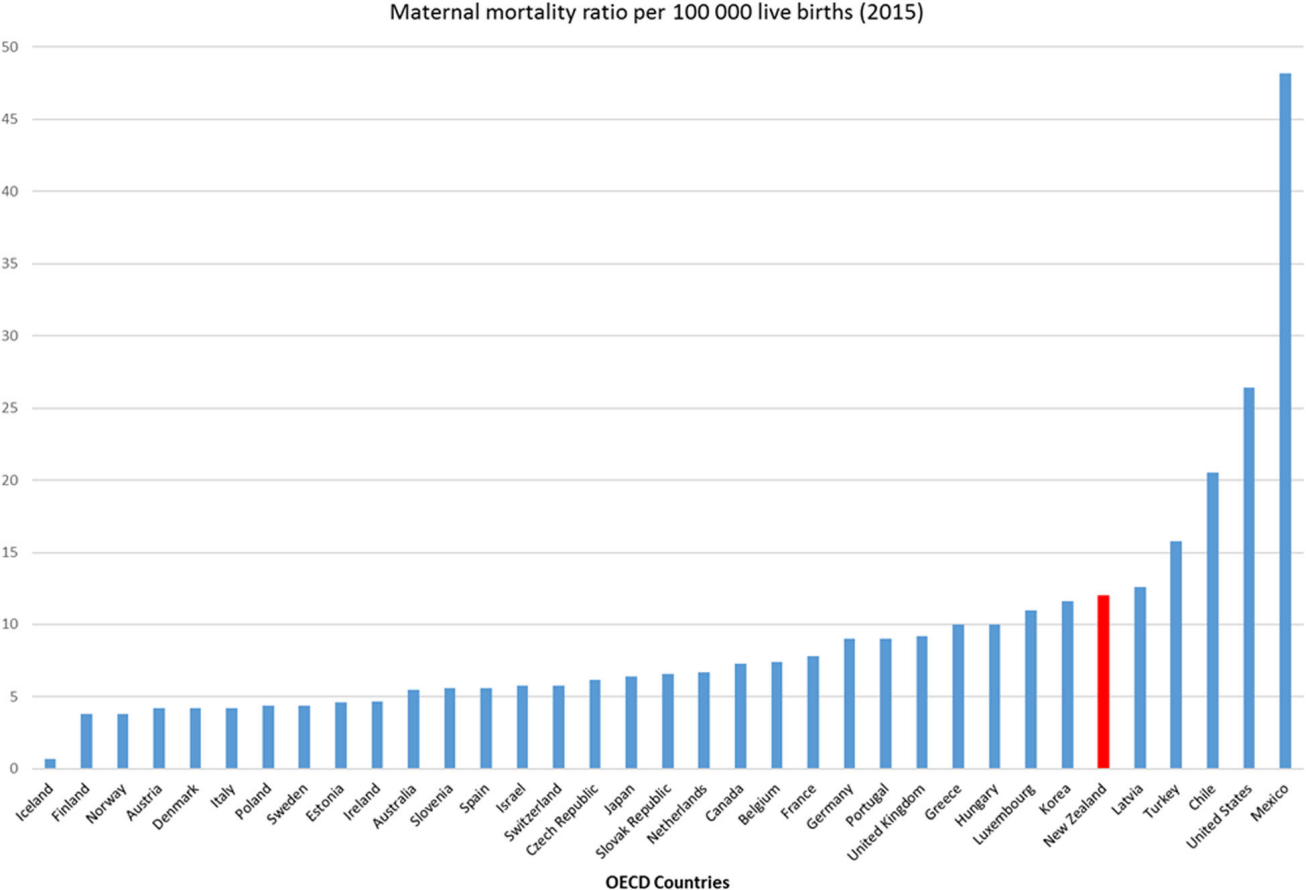
Maternal mortality ratio per 100,000 livebirths (2015) Data adapted from OECD report [8]

Although maternal mortality is a relatively rare event in New Zealand [18], the 2015 rate was 12 per 100,000 births while neighbouring Australia was 5.5 per 100,000. Figure 1 shows New Zealand’s ranking within OECD countries based on 2015 data [8].

**Fig. 2:**
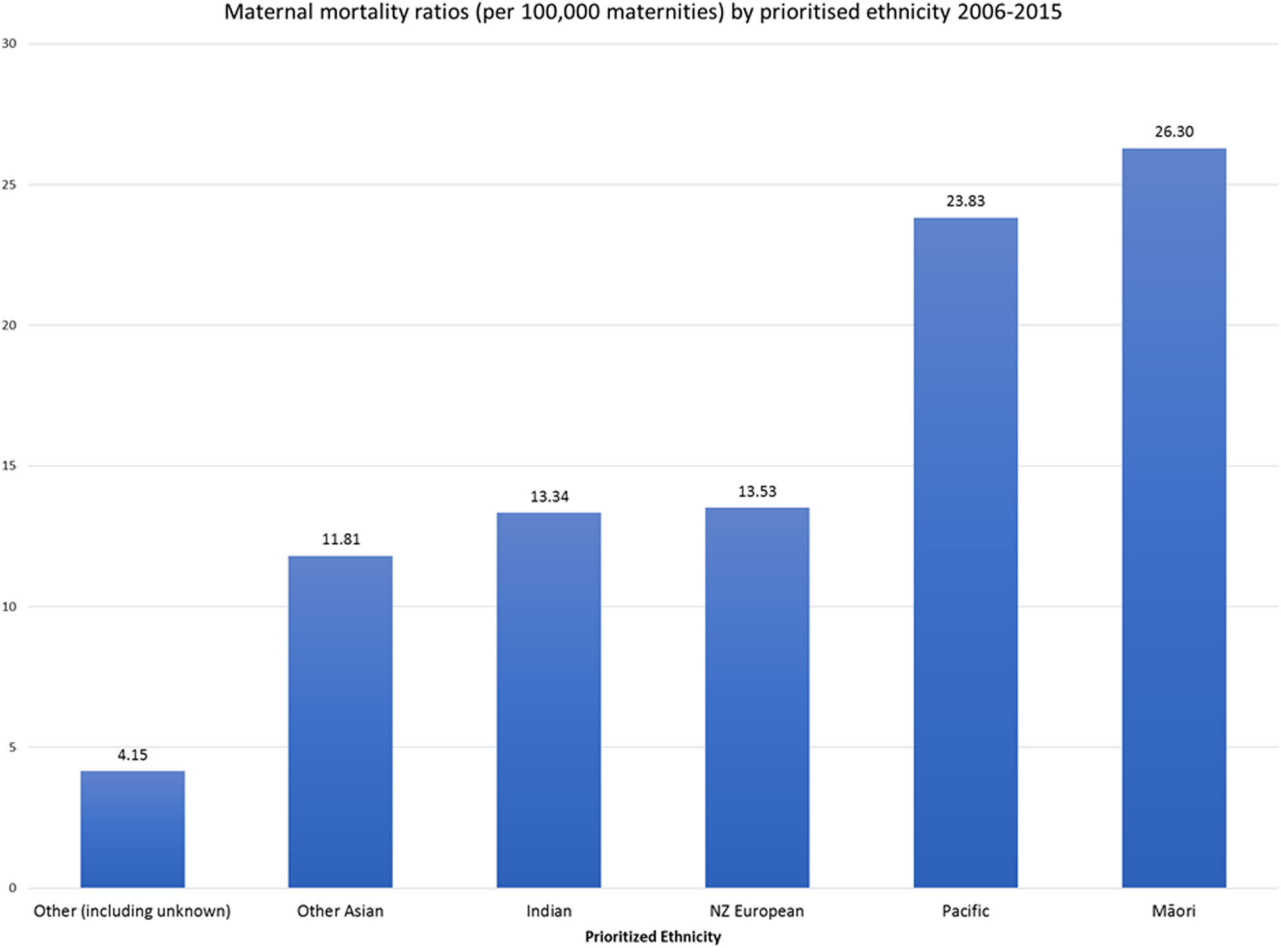
Maternal Mortality Ratios in Aotearoa New Zealand (per 100,00 maternities) 2006–2015. Data adapted from PMMRC report [19]

What Fig. 1 does not show is the inequity within the New Zealand population, for instance there is a significantly higher maternal mortality rate among Māori (26.3/100,000 maternities) and Pacific women (23.8/100,000 maternities) compared to New Zealand European women (13.5/100,000 maternities) in combined data from 2006 to 2015 [19] (Fig. 2). Similar disparities are evident in this report in data around perinatal death.

Mortality statistics are often cited as “the tip of the iceberg” (p.794 [20]) when examining inequity in outcomes for birthing women. A paradigm shift from solely prevention of maternal death to promoting women’s health and wellness within the World Health Organization (WHO) has resulted in new frameworks to address these issues [21]. In New Zealand, maternal morbidity studies have shown similar trends to maternal mortality [22–26] and highlighted inequity issues for the most deprived New Zealand women and over-representation of poor maternal outcomes for Māori and Pacific women [19, 26, 27].

Rumball-Smith [28] analysed the relationship between maternal outcomes and process of care, including rates of caesarean section, induction of labour, instrumental vaginal delivery, and epidural analgesia. Māori women all had lower intervention rates despite being at higher risk of poor outcomes in pregnancy. Rumball-Smith posits that “it is possible that the disparate rates in the studies above [29, 30] represent overuse of services by non-Māori, as opposed to underuse in Māori” (p.96 [28]) and also suggests that research indicates Māori women receive less clinically acceptable care.

Another indicator of maternity system equity comes from canvassing the experiences of women. The most recent assessment of maternity consumer experience via a postal survey by the Ministry of Health of all women who had live births between December 2013 and February 2014, had a low overall response rate of 29.4% and even lower proportions of, Māori (18.4%) and Pacific women (14.5%) [31]. There was no apparent difference in overall satisfaction by ethnicity, but with margins of error such as these it is difficult to be confident in the data; it is clear that fewer women from two ethnic groups with worse maternal outcomes responded. Preliminary analysis of this dataset and scrutiny of latent equity barrier variables (physical access, service cost and cultural factors) indicate that highly deprived women are disadvantaged by physical access factors and women in remote rural locations pay more for their care unrelated to travel costs [31]. which may indicate skewed health facility and resource distribution.

In sum, despite a socialised health system, maternal inequity persists in New Zealand. These disparities in maternity care and outcomes, system of care indicators and maternal satisfaction are, on the surface, difficult to explain. This review aims to examine the ‘causes of the causes’ of maternal inequity specific to New Zealand, and explain factors underlying continuing disparity, despite a free, women-centred, continuity of care maternity system.

## Methodology

**Fig. 3:**
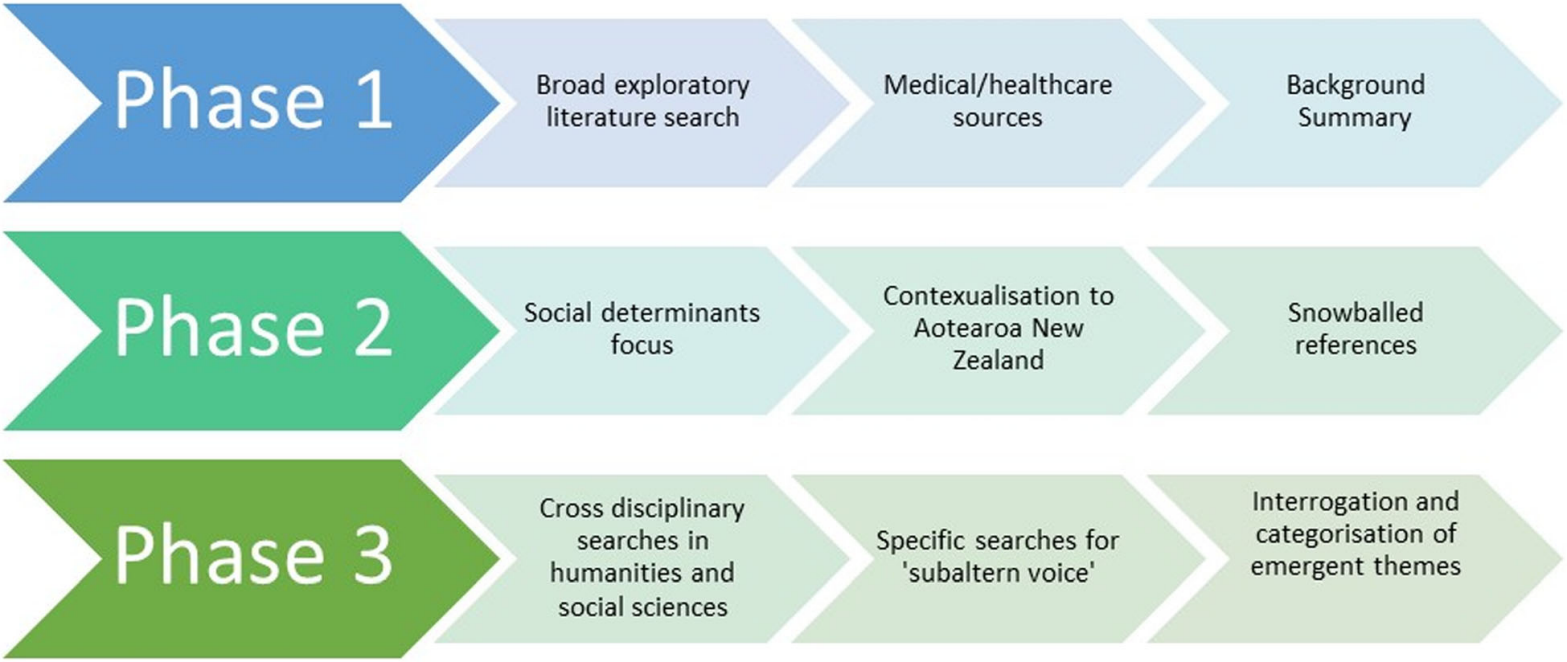
Phased Methodology of the Review

An integrative methodology [32, 33] was used to locate, compile, review and synthesise literature in a phased and iterative approach. This methodology can be conceptualised as developing “a more comprehensive account of a specific phenomenon or relationship than of the related basic research reports separately” by integrating both theoretical and empirical works [33]. Thus, the method allowed scope to draw from a variety of sources including quantitative, qualitative and grey literature to produce a comprehensive evaluation of the ‘causes of the causes’ (or social determinants) of maternal health inequity, with a focus on New Zealand. This approach was appropriate for the cross-disciplinary and disparate knowledge sources being examined, i.e. social and scientific research, historical, structural, governmental, economic and policy documents. The phased nature of the review process is illustrated in Fig. 3.

### Selection of data

The broad scope of this structured integrative critical review (i.e. inclusion of theoretical and empirical works, and multiple research designs) meant that selection was complex but the wide sampling frame added to the depth of the review and allowed for a rich narrative analysis [34]. The three phases of searches returned over 650 relevant articles. For empirical research, selection was informed by a quality evaluation and rating based on methodological rigour, and authenticity, informativeness, and representation [32]. For instance, an international randomised trial of a maternal health intervention to address inequity might rate highly for rigour and authenticity, but low for informativeness and representation. In contrast, a survey in New Zealand using Kaupapa Māori methods (an indigenous methodology where research and evaluation is done by Māori, with Māori and for Māori [35]) collecting data from Māori women might rate highly in all four categories.

#### Iterative searching

To enable a broad exploration of international literature the initial search employed Boolean AND/OR operator combinations of Phase 1 keywords using medical and healthcare related databases i.e. PubMed, Scopus, Embase and CINAHL (Table 1). Having read and summarised the background equity data on comparative international rates of maternal morbidity and mortality, phase 2 search terms were introduced and combined with the phase 1 keywords to focus on New Zealand, and social causes of inequity. At this point, searching was still confined to medical and health databases. In phase 2 additional references and search terms were located though a snowball technique [36] utilizing literature reference lists and “cited by” functions of databases (Fig. 3).

**Table 1:** Search Strategy – Phases of search terms

Phase 1 (Background)^a^	Phase 2^b^	Phase 3^b^
Maternity/al	Policy	Colonial/ism
Birth	Services	Feminism
Obstetrics	Planning	Cultural Safety/Competence
In/Equity	Access	Social Justice
In/Equality	Social Determinants	Neo-Liberal
Health	Capability	Indigenous
Disparity	New Zealand	Māori
Outcomes	Continuity	Pacific/Pasifika
Morbidity		Racism/Race
Mortality		Universal Health Coverage/Socialised Health
		Minority
		Poverty/Deprivation
		Vulnerable

^a^ last 10 years; ^b^ no date restriction

As thematic analysis of phase 2 literature sensitised the first author (PD) to social determinants particularly relevant in the New Zealand context (e.g. ongoing impact of colonialism), phase 3 keywords were introduced to interrogate the emergent themes further. At this point specific searches and chain-referral - using the University of Otago Ketu library search system and Google Scholar - were undertaken to find primary sources of the ‘subaltern voice’ [37–39], which are sometimes difficult to locate in common indexes and represent infrequently heard voices in the academic literature (Fig. 3). This was particularly important in the areas of colonialism and cultural factors. Examples of relevant articles located during this phase were found in ‘Pimatisiwin: A Journal of Aboriginal and Indigenous Community Health’ [40] and ‘AlterNative: An International Journal of Indigenous People’ [41, 42]. Additional relevant material was found in education, economics, political, anthropology journals and other humanities and social science sources. Phase 3 also interrogated the interconnectedness and links between themes.

#### Analysis

This review was informed by the World Health Organization (WHO) Social Determinants of Health (SDH) framework as outlined in a 2010 discussion paper [43]. This provided a model for phase 2 literature searching (see above), and also the coding of data into categories which aligned with the three elements of the SDH framework (socio-economic and political context, structural determinants and socioeconomic position, and intermediary factors). Data within categories were then inductively analysed for social the underlying causes of inequity [44]. In an iterative process each item within every category was compared with others, to generate themes that represented the identified social determinants; diagramming was used to visualise the interaction between themes. The themes were compared back to the WHO Social Determinants of Health framework for external validity, and cross-disciplinary data sources were then specifically sought to interrogate the interactions and for comparison with the framework. Coding and analysis were conducted manually with data compiled in Excel spreadsheets.

#### Findings

**Fig. 4:**
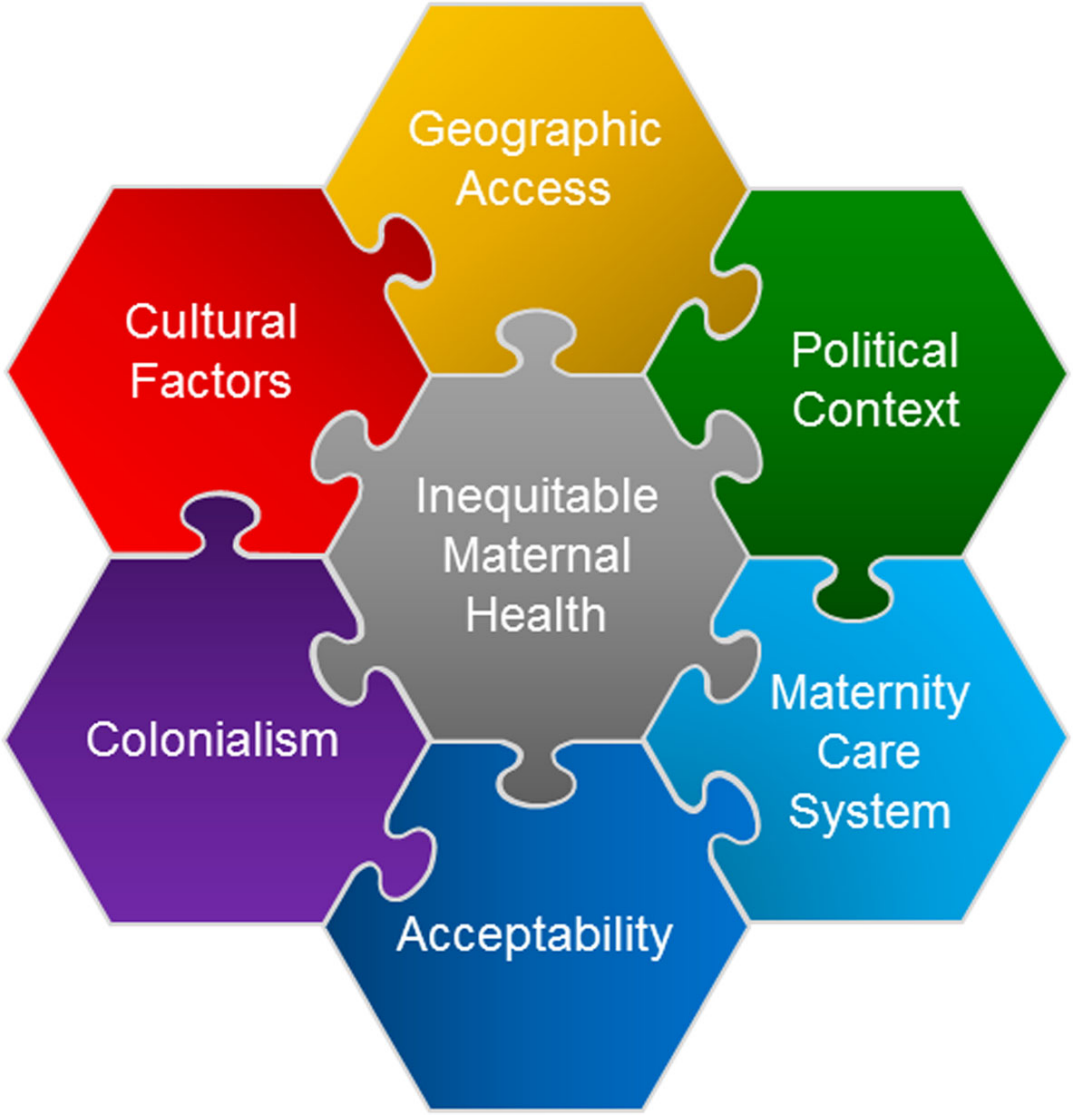
Barriers to Equitable Maternal Health in Aotearoa New Zealand

The literature review was completed in the last quarter of 2018. Analysis identified six factors that underpin maternal health inequity in New Zealand: geographic access, political context, maternity care system, acceptability, colonialism, and cultural factors. The interrelatedness was obvious, and boundaries between factors were blurred in places, so an interlocking conceptual model was developed to represent the complexity of connections (Fig. 4). The connectedness of factors means that, acting together, they have a cumulative effect beyond what each has alone. Greater exploration of each factor is given below.

#### *Geographic Acce*ss

In the analysis presented, geographical access was separated out from the five aspects of access (approachability, acceptability, availability and accommodation, affordability, and appropriateness) as defined by Penchansky and Thomas [45] and this is in contrast with the broader perspectives of access taken by some researchers [46]. The literature indicated that there were strong differences in how this broad definition of access affected specific groups. Distinguishing between geography and other aspects of access has generated a more nuanced analysis and recognises the population distribution in New Zealand with a few concentrated urban areas and also large sparsely populated regions.

Early in equity research, there was a focus on physical accessibility of health care services in general and this theme dominated much of the literature. Hart’s Inverse Care Law “the availability of good medical care tends to vary inversely with the need of the population serviced” (p.405 [47]) held true at the time and so systems moved to providing services to high need communities to solve spatial inequities. Geographic inequity is one of Whitehead’s principles for action “making high quality health care accessible to all” (p.440 [48]) and ensuring services are geographically linked to need.

When applied to maternity care, the concept of access tends to be measured by distance to birthing facilities and what kind of clinicians, services and facilities are available. Timing of initiation of antenatal care in pregnancy which is associated with poorer maternity outcomes [49–52], is a proxy often used to measure physical access. However, this could be associated with other socio-economic or system factors unrelated to where a mother might live.

While there are several medium sized urban centres in New Zealand, there are large areas of low-density rural population. Since the 1990s, neoliberal reform of the health system has stripped services from rural areas and so increased access barriers [53–56]. Rural maternity units closed and distances to any maternity facility increased for rural women [57]. These closures continue; for example, the Lumsden Maternity Unit closing in April 2019.

Studies examining the use of remaining primary units as birth place in New Zealand [58, 59] show that many rural women are choosing to travel to base hospitals with secondary services in order to access specialised care such as epidural anaesthesia and avoid transfers in labour [60–62]. Travel for birth has costs associated with it and rural women may also need to travel to main centres for specialist antenatal care in the case of pregnancy complications as recommend by the New Zealand guidelines for referral. These Guidelines outline criteria and process for when care level changes from primary to secondary/tertiary [63]. Thus, health system structure – in particular where care is delivered – decreases access for rural women.

Urban women may also have difficulty with physical access to available services, and social issues such as problems with child care, accessing transport and having to attend clinics rather than having home visits may be experienced by rural and urban women [64, 65]. Home visits are no longer the usual mode for primary antenatal care, and requiring specialised care due to complications can mean even greater physical access difficulty for urban women where it is possible that public transport to attend a tertiary clinic could take hours. Lee & North [66] reported that Māori sole mothers prioritised their children’s needs over their own health care which created an additional barrier e.g. child care might be necessary to attend clinics or parenting responsibilities could make travel to attend services more onerous.

New Zealand has acute maternity work force shortages [67] and this is severe in rural areas [68]. The problem has spread to larger urban centres as many midwives leave the profession [69–72]. Thus, geography where there is no or limited local service available due to staff shortage, in turn restricts choice of carer, disadvantaging some women. For instance, Makowharemahihi et al. [73] found that young Māori women would give up looking for a midwife if calls were not returned, or the first midwives they approached had full case lists. This in turn led to late booking – a risk factor for a poor outcome in an already vulnerable group – and thus underlying cause of the late booking may be social, structural or both.

#### Political factors

The CSDH categorizes political context as an intermediate structural determinant of health in their framework [3]. Backfield & Kreiger systematically reviewed the relationship between political factors and health inequity concluding that there are four main factors “1) the transition to a capitalist economy; 2) neoliberal restructuring; 3) welfare states; and 4) political incorporation of subordinated racial/ethnic, indigenous, and gender groups” (p.155 [74]). We suggest that all of these are evident in New Zealand [75, 76].

Backfield and Krieger’s work alludes to the transition to a capitalist economy in former communist Eastern bloc countries in Europe. However, this can be extrapolated to New Zealand, where colonial occupation replicated an industrial capitalist economy, with dramatic effects on traditional Māori ways of life and living (called tikanga). This shift and ensuing land loss had long lasting effects on the indigenous population and is further explored in the section on colonialism.

Chronologically, another major political change occurred in New Zealand with the introduction of the Social Security Act in the 1930s, and universal medical benefits in 1941. Secondary healthcare (and maternity care) became free, and the ‘welfare state’ was introduced. Theoretically, a welfare state and universal health coverage should reduce if not eliminate health inequities. However, analysis of longitudinal data in New Zealand show that inequity persists; for example, in both overall population life expectancy and maternal mortality by ethnicity [19, 77, 78]. Persistent inequity in a welfare state is not particular to New Zealand, with international research demonstrating that health inequalities persist and in some cases worsened [79]. Mackenbach describes this persistence as a ‘meta phenomenon’ made up of a complex mix of individual, structural and cultural factors. He suggests that health status feeds social inequity, as health can be a defining factor in socioeconomic status [79, 80].

Next came the neoliberal reforms of the 1980s, when New Zealand was subject to extensive neoliberal economic, social and political change. Some described this as the “New Zealand Experiment” [81] as such extensive implementation of neoliberal policy was not seen in other democratic countries [82]. Characterized by belief in economic growth as the means to achieve social progress, and minimal state intervention in economic and social affairs, the consequences of neoliberalism in New Zealand were rising unemployment, labour casualisation, increased part-time work, and an uncertain labour market [83]. The gap between Māori and non-Māori in health, income and education widened [81, 84] and has not reduced, with persistent poorer health status and outcomes for Māori. These inequalities continue with the wealth gap increasing [85].

The negative health impact of widening income inequality consequent on neoliberal policy is seen globally [86] and is detailed in work by Wilkinson & Pickett [87, 88] and Marmot [89]. Poor health under neoliberalism is viewed as personal failure [87, 90]. This is largely to do with the neoliberal philosophies of running a country like a business and the principle of individualism, where ideals of public good and community are replaced by an expectation of self-responsibility [86, 91]. In this model, patients become consumers of health services and policy becomes product [92].

New Zealand is in a late colonial period (see Late Colonialism) and is still grappling with truly honouring the principles of the Treaty of Waitangi in policy and practice. New waves of diverse immigrants are adding additional layers of complexity. Racism is widespread yet not broadly acknowledged [93–97]. At a structural and policy level it appears that real acknowledgement and addressing this systemic racism is slow. Chin et al. state that “structural racism, colonialism, white privilege and implicit biases” has yet to be addressed fully in the New Zealand health system (p.849 [98]).

One side effect of neoliberal policy that could be viewed as positive was the Nurses Amendment Act 1990 which enabled midwives to work as autonomous practitioners running their own businesses as contractors to the Government. While this was very liberating at the time, the contract under which they work (Section 51, then Section 88 of The Act) controlled pay, and did not allow for inflation or increased cost of living/business costs. The statute had to be amended constitutionally for any change in payment rates. Under the guise of ‘choice’ it pitched midwives into competition with each other - and the remaining General Practitioners offering obstetric care - for ‘clients’. Also, midwives could not charge a co-payment for services, but this was and is an expectation for private obstetricians who were allowed to charge additional fees under the Act. At the time of writing midwives were campaigning for a redesign of the funding system to reflect the change in their work loads, acuity, business costs, need for locum cover, rural cost and other factors [99].

#### Maternity care system

The Lead Maternity Carer Model in New Zealand is led by primary care midwives, providing continuity of care within a partnership model [100]. The midwifery partnership model is aligned to the principles of partnership, protection and participation in the Treaty of Waitangi [101, 102]. As outlined previously, this system is unique in the international context and so comparisons to other systems are difficult [12]. In theory, a system based on partnership and individualised continuity of care should provide a framework that is capable of working to ameliorate inequity, however it is only one of the inter-related factors associated with equity. One reason some models of care cannot be relied on in practice as a mechanism for equity, is that the clinician/patient relationship cannot be equal due to an underlying power imbalance. This is often exacerbated when the clinician is from a dominant and privileged group and the patient from a vulnerable population [41, 103–105].

The change from General Practitioner led to the Lead Maternity Carer (LMC) midwifery focused system In New Zealand was congruent with the 1970s feminist movement demand by women for women-centred care and a rejection of the medical model as the basis for maternity care [106, 107]. The midwifery partnership model works towards addressing clinician/patient power differences. However, systemic issues leading to workforce shortages mean women have fewer midwives to select from – making it more difficult to exercise choice – and in some localities it can be difficult find any LMC at all [69, 72].

In addition equitable care is constrained by the structure of the maternity system that has inadvertently led to a workforce that is not easy to mobilise to meet geographic areas of demand, has issues of under-funding and resourcing, while acuity and social demands increase [72, 108, 109]. Achieving equity for women with complex needs is particularly problematic in the current system, often concentrated in particular geographies, e.g. higher proportions of lower socio-economic groups in some localities. If complex clinical needs require secondary and tertiary level maternity services, which remain free, access can be more difficult as these services are centralized to regional urban centres. Issues may arise in the transfer between primary care and care, as women experiencing physical access issues find it hard to attend distant clinics while primary care is usually available closer to home [110]. Successful consultation and transfer is also reliant on good interdisciplinary relationships [111].

Wrap around care initiatives for vulnerable populations with complex maternal needs have been piloted. For example, following an external maternity services review [112] in response to Counties Manukau District Health Board having higher perinatal mortality rates than the rest of New Zealand, a complex care outreach programme was established. While attempts to improve integration between primary and secondary services in other health fields has shown limited effect on clinical outcomes, these initiatives showed high patient satisfaction and improved process and service delivery outcomes [113].

#### Acceptability

Clear communication, ease of engagement, being listened to, being treated with compassion, dignity and respect in a non-judgemental way and continuity are indicated as making maternity services more acceptable to women and therefore better utilized [50, 114, 115]. Women’s engagement with a service is often linked to their maternity outcomes [22, 116, 117].

A meta review by Nair et al. of barriers to quality care improvement in maternal and neonatal health found that acceptable/patient centred care was a key indicator for quality. Several studies overseas and in New Zealand found that navigating the system to engage with care was challenging [73, 118] and some found a variety of barriers including cultural ones [65]. Cultural factors were also identified in Makowharemahihi’s study of young Māori women [73] regarding their engagement with the maternity care system and other studies found a variety of barriers including cultural ones [64, 65].

Choice of [maternity] carer is a key concept in acceptability [45]. The current maternity system is largely ‘one size fits all’. Most women choose a midwife as their lead maternity career (LMC) as alternatives are few; there are almost no General Practitioners providing obstetric care in New Zealand and fee for service obstetrician care is costly and not widely available. For a vulnerable woman finding the right person to lead their maternity care can be critical for achieving the best outcomes [115, 118]. Māori and Pacific women frequently express desire for a midwife of their own ethnicity/culture; reasons for this include cultural compatibility and understanding, and a more equal partnership with the midwife [40, 41, 64, 119–121].

Most women responding to The Consumer Satisfaction Survey series are satisfied by their maternity care, with 77% being very satisfied or satisfied in the 2013/14 period, albeit with low response rates especially for Māori and Pacific Women [31]. These surveys tell us how women experience the current system, but not what they value or want from it. Various initiatives have increased consumer involvement in all aspects of maternity systems and governance, such as having consumer representatives on Ministry of Health maternity guideline development groups and The Perinatal and Maternal Mortality Review Committee, but research and implementation of Patient Reported Experience Measures (PREMS) and Patient Reported Outcome Measures (PROMS) is lacking.

#### Late colonialism

Health inequity cannot be addressed without consideration of colonial history, Treaty obligations and the legacy of historic trauma [122]. The Māori proverb ‘*Ka mua, ka muri*’ which translates as ‘looking back in order to move forward’, also illustrates the importance of acknowledging historical context in any survey of inequity in New Zealand.

New Zealand is a former British colony. The continuing inequities in general and maternal health of Māori and Pacific women are indicative of the ongoing effects of this problematic history. The final report of the Commission on the Social Determinates of Health acknowledges the unique inequities faced by indigenous peoples and states that “Colonization has deterritorialized and has imposed social, political, and economic structures upon Indigenous Peoples without their consultation, consent, or choice … As such, Indigenous Peoples have distinct status and specific needs relative to others” (p.36 [3]).

Much has been written about the connection between colonial history and health inequity, most of it on the high income former colonies in the Canada, Australia, New Zealand, and the USA (CANZUS) group [123]. This group represents former British colonies where indigenous peoples were severely affected with introduced diseases decimating population, loss of self-governance, land, and language. Massive upheavals of lifestyle and social cohesion and near loss of culture and traditions were compounded by social change due to urbanization in the 1950s and neoliberal reform in the 1980s.

New Zealand is unique in the CANZUS group with founding documents (signed by representatives of the British Crown and Māori) which form part of the country’s constitutional law. Following conflicts between the indigenous Māori population and European traders and settlers, in 1840 a formal agreement was made between Māori and representatives of the Crown. The Treaty of Waitangi/ Te Tiriti o Waitangi, New Zealand’s founding document, was intended to be a partnership between Māori and the British Crown. While its aim was to create unity, differing interpretations of the Treaty, and breaches of it, have caused ongoing conflict and tensions [124, 125].

Post-Treaty, European population numbers rose quickly, very soon outnumbering Māori. With this wave of settlers came the need for land and in 1845 the New Zealand Wars broke out resulting in vast areas of land being confiscated from the Māori as punishment by the government under the New Zealand Settlements Act in 1863. Māori have a deep spiritual relationship with the land which is exemplified in the word *whenua* which means both land and placenta – both supporters of life. Connection to the whenua underpins the holistic approach to Māori health [126, 127]. Land confiscation following the New Zealand Wars has had an ongoing and generational detrimental effect on Māori health [94, 121, 128–130]. While Treaty settlements have paid iwi^1^ some financial recompense, land has not been restored and nor, arguably, has the identity and wellbeing associated with the land [131].

An ongoing series of legislations and responses between Māori and the settler population means that all peoples of New Zealand are still grappling with the meaning and legacy of colonial New Zealand.

The large resident Pacific population also needs to be acknowledged as partially due to remnants of British colonialism in the Pacific, and the former and ongoing political relationships between the two. From the Pacific, New Zealand is seen as an attractive destination to pursue educational and work opportunities and there has been strong immigration from Pacific nations since the 1960s [132]. In 2013, 7.4% of the population of New Zealand identified as Pacific, with two thirds of these living in the greater Auckland area [133].

The ongoing effects of this colonial history, of loss, displacement, and marginalisation cannot be underestimated when considering equity however increasing social progress and self-determination movements are working towards improvement [134].

#### Ethnic, cultural, and race issues

In New Zealand there are distinct obligations to tangata whenua^2^ under the Treaty of Waitangi that need to be honoured. The basic principles of partnership, participation and protection should pervade all aspects of healthcare and the Ministry of Health has made a commitment to this in its strengthened He Korowai Oranga Māori Health Strategy [135]. However, Came et al. [136] have questioned real commitment to this programme in the 2016 Health Strategy and true engagement with Te Tiriti at a structural systems level.

One way to begin to address barriers to equity in ethnic groups is acknowledgement and utilization of locally developed health care models. One example is the Te Whare Tapa Whā model which was developed by Durie [127, 137] and incorporates four aspects of wellbeing: Te Taha Hinengaro (psychological), Te Taha Wairua (spiritual), Te Taha Tinana (physical), and Te Taha Whānau (family). This model is not without its critics and Heaton has recently expanded on the model expressing that it is missing the foundation that whare^3^ are built on – whenua (the land) which is also key to Māori wellbeing [126]. Designed to be dynamic, such models appear to be suitable and acceptable basis for the advancement of health and promoting health equity. However, it is difficult to quantify aspects of application or attainment because of how and what health data are collated by Government departments and agencies.

Incorporating values important to ethnic/cultural groups reflects the principles of the Treaty of Waitangi but also that of patient participation promoted by Whitehead [48] and the CSDH [3], Step Six of the WHO Innov8 ‘leave no one behind’ programme states “participation is a core principle of a human rights based approach, and intersectoral action is implicit in the nature of the right to health as an inclusive right that includes a wide range of underlying determinants that influence health” (p.151 [138]). These are somewhat idealist models and to really address ethnic health inequity, the ‘elephant in the room’ has to be addressed; the barrier of racism. An international meta-analysis of 105 studies by Carter & Lau [139] found “a statistically significant effect size between racial discrimination and health” (p. 232). Another systematic review revealed similar results [140].

New Zealand’s colonial legacy includes strong elements of racism and discrimination with an ongoing cultural malaise that perpetuates inequities in the health system [93, 141–144]. There is no doubt that ethnicity, specifically identification as Māori, is a determinant of health inequity. In their survey analysis, Cormack et al. write that “Discrimination was associated with poorer self-rated health, poorer mental health, and greater life dissatisfaction” of Māori and other minority ethnic groups (P.1 [93]). Heather Came [143] has written extensively on racism in New Zealand institutional public health and policy settings. Ricci Harris [141, 145, 146] has first authored several papers linking racial discrimination and health in New Zealand, finding racism is “significantly associated with poor or fair self-rated health; lower physical functioning; lower mental health; smoking; and cardiovascular disease” (p.1428 [146]).

Throughout the findings, repeated examples of inequity (including inequity in maternal health) associated with Māori ethnicity are cited. Some research has specifically linked inequity (unjustness) with unfairness and racism. For instance, an analysis of data from the Growing Up in Aotearoa New Zealand longitudinal study, found that “Māori, Pacific, and Asian women who had experienced unfair treatment by a health professional in their lifetime were 66 % more likely to suffer from postnatal depression” (p.1 [95]). In another study, researchers recruited women from antenatal clinics to investigate prenatal stress, and cortisol reactivity in their newborns and concluded that “Women reporting [racial] discrimination experience had worse self-rated health, higher evening cortisol and gave birth to infants with higher cortisol reactivity” suggesting racism may have “biological impacts in pregnancy and across generations” (p.36 [147]). Studies in other nations have found similar maternal impact in their indigenous populations [148–152].

### A way forward

Theoretical models have been proposed such as The World Health Organization’s *Closing the Gap* and *No one Left Behind* programmes to address social determinants of health in line with the Sustainable Development Goals. In New Zealand, a renewed commitment to health inequities has been signalled in the Ministry of Health’s equity work programme. A new definition of equity was signed-off by the Director-General of Health in March 2019 as follows:

“In Aotearoa New Zealand, people have differences in health that are not only avoidable but unfair and unjust. Equity recognises different people with different levels of advantage require different approaches and resources to get equitable health outcomes.” [153]

It is also observed that local advancement in the area of Patient Reported Outcome and Experience measures would likely add to the knowledge of (un) acceptability of the maternity system (and links with engagement), and what is important to women in Aotearoa New Zealand in their maternity journey.

While carrying out this literature review a number of enterprises were identified that appear successful in practice at promoting equity. Locally developed and based programmes, many of which are dynamic and innovative and with real participation and partnership with communities and health consumers are achieving results. Particular effect has been noted in programmes run by Māori Health providers and also research involving community based and developed interventions. In maternity related work, Glover successfully trialled a smoking cessation intervention for pregnant Māori women delivered by ‘Aunties’^4^ [154] and a very successful sudden unexpected death in infancy (SUDI)^5^ intervention based on an indigenous sleep device (wahakura) has had promising results [155]. These projects are notable in that they are led by indigenous researchers, are strongly engaged with the communities they seek to help and utilize kaupapa Māori methodologies. Such participatory approaches are consistent with global guidance, such as steps 6 & 7 of the CSDH Innov8 programme which aims to “formulate recommendations to redesign the programmes … redesign commences with identification and prioritization of the changes required to consider the contextual circumstances and differential needs of the prioritized subpopulation, tackling the barriers they face and, most importantly, addressing the mechanisms that explain inequities in programme results.” (p.12 [138]).

## Conclusion

This review has identified a network of enmeshed structural and systemic factors at play in New Zealand that contribute to ongoing maternal heath inequities as measured by outcomes, systems of care, and women’s experiences. While many of the factors are identified in the international literature, these features are grounded in unique historical, cultural and political circumstances specific to New Zealand, resulting in a cluster of inter-related social determinants that create barriers to equitable outcomes for mothers and babies. Research with a primary focus on equity is unique in the maternity care literature in Aotearoa New Zealand; research to date has only established the existence of outcome inequities. This review shows that social determinants and equity warrant further scrutiny, and the review suggests specific domains for investigation.

Due to the complexity of the underlying issues, it is suggested that a remedial path will not be easy [98]. It is likely that an equally complex web of factors would be identified in any country where the structural causes of health inequity are examined.

The interrelated and potentially compounding aspects of the six factors identified means no one single solution can be applied as an intervention to eliminate maternal inequities. To this end, there may even be negative impacts of interventions addressing just one factor. Holistic approaches addressing structural and intermediary barriers, or suites of solutions - for example wrap around care programmes - need to be considered and in identifying inequity reduction programmes that appear to be working, common threads of self-determination and community participation are key. Herein may lie a way forward - broad programmes and policies as suggested in the WHO literature, contextualised for specific country and community, developed and implemented in a truly collaborative process, and, in the context of Aotearoa New Zealand, honouring the principles of Te Tiriti O Waitangi.

## Data Availability

Not Applicable (review).

## Abbreviations

CSDH: The Commission of the Social Determinants of Health
LMC: Lead maternity carer
MMR: Maternal mortality rate
NZ: Aotearoa New Zealand
OECD: The Organisation for Economic Co-operation and Development
PMMRC: The Perinatal and maternal mortality review committee
PREMS: Patient reported experience measures
PROMS: Patient reported outcome measures
SDOH: Social determinants of health
WHO: World Health Organization

## Footnotes

1 Set of people bound together by descent from a common ancestor or ancestors; literally: bone; modern meaning: tribe (132)

2 local people, hosts, indigenous people - people born of the whenua, i.e. of the placenta and of the land where the people’s ancestors have lived and where their placenta are buried. In Aotearoa New Zealand – Māori.

3 House/home

4 Widespread cultural term meaning a woman who may or may not be related but has community standing. Often older and provides guidance and assistance.

5 Māori post-neonatal mortality is 3 times that of non-Māori. Māori place considerable cultural value on bed-sharing and also have high rates of maternal smoking (53% vs 8%) (151)
